# Paediatric femur fractures—the value of contextual information on judgement in possible child abuse cases: are we bias?

**DOI:** 10.1007/s00431-020-03704-6

**Published:** 2020-06-17

**Authors:** Marie-Louise H. J. Loos, Wies M. Allema, Roel Bakx, Reinoud D. Stoel, Rick R. van Rijn, Wouter A. Karst

**Affiliations:** 1 Department of Paediatric Surgery, Emma Children’s Hospital, Amsterdam UMC, University of Amsterdam & Vrije Universiteit Amsterdam, Meibergdreef 9, 1105 AZ Amsterdam, The Netherlands; 2 Division Specialist Services and Expertise, Team Forensic Statistics, Netherlands Forensic Institute, PO Box 24044, 2490 AA The Hague, The Netherlands; 3 Department of Forensic Medicine, Section on Forensic Paediatrics, Netherlands Forensic Institute, PO Box 24044, 2490 AA The Hague, The Netherlands

**Keywords:** Child abuse, Influence, Femur fracture, Contextual information

## Abstract

The majority of paediatric femur fractures result from accidental trauma; however, it is important to consider non-accidental trauma, especially in pre-ambulatory children. We study whether irrelevant contextual information subconsciously influences conclusions of healthcare professionals with respect to whether observations provide evidence for non-accidental trauma. A survey with nine radiographs of femur shaft fractures was designed. Two different clinical histories (vignettes) with contextual information were designed, non-abuse versus abuse context. One of both vignettes was randomly assigned to the radiograph shown to the participant, followed by a question with a 5-point answer scale, which represents a verbal expression of the likelihood ratio of the fracture regarding a non-accidental versus accidental cause. Participants were medical residents and staff members of different specialties from several Dutch hospitals. A total of 172 participants responded. The reported evidential strength of the vignettes with a non-abuse context was 0.19 (*n* = 784; 95%CI 0.10–0.28) and for the abuse context 0.94 (*n* = 764; 95%CI 0.86–1.02; *p* < 0.001). Women reported a stronger evidential strength than men, but both were influenced by context. Emergency department and paediatric doctors were more likely to decide that non-accidental trauma was the cause; paediatric radiologists were the least likely. Experience in years of practice and current function did not prevent participants from being bias.

*Conclusion*: This study shows that the interpretation of medical results by healthcare professionals can be influenced by contextual information, such as low income and marital status, which are irrelevant to the decision as to whether abuse might have occurred. Given the same information about an injury, women, emergency department and paediatric doctors were more likely to decide that non-accidental trauma was the cause, while paediatric radiologists were least likely to decide this outcome. It is important to prevent contextual influence as much as possible, by recognizing it and implementing a management contextual information procedure.

**Table:** 

**What is Known:** *• Contextual information is of possible influence on healthcare professionals in identifying non-accidental trauma.* *• Increased working experience is thought to be protective against this influence.*	
**What is New:** *• Contextual information influenced the interpretation of medical results by healthcare professionals regardless of work experience.* *• The interpretation of medical results by healthcare professionals is influenced by both affirmative and negative contextual information.*

**Electronic supplementary material:**

The online version of this article (10.1007/s00431-020-03704-6) contains supplementary material, which is available to authorized users.

## Introduction

The majority of paediatric fractures result from accidental trauma such as traffic accidents, falls or while playing sports.[1, 2] Nevertheless, in 25–56% of young children (< 1 year), fractures are caused by non-accidental trauma.[2] All healthcare professionals treating children in their daily practice should be aware of this cause, especially in pre-ambulatory children, [3, 4] and they should be able to recognize ‘red flags’ linked to non-accidental trauma.[5] The child’s age, level of development, type of fracture and fracture location are main indicators that show whether or not the child suffers from non-accidental trauma.[1] Children with femur fractures are often referred for evaluation to Child Abuse and Neglect teams (CAN), due to the high prevalence of reported non-accidental femur fractures among young children, ranging from 16.7 to 35.2% (< 12 months old) versus 1.5 to 6.0% for older children.[4] Additional risk factors of non-accidental femur fractures have a suspicious history, non-ambulatory status and presence of additional injuries on physical examination. Fracture morphology and fracture side are not associated with non-accidental trauma. [4] Especially because classification depends on the experience of healthcare professionals.[6] In other words, different healthcare professionals may classify the same femur fracture differently. In addition, Pandya et al. showed that in the case of young children (< 18 months old) with femur fracture, the odds ratio of abuse is 1.8, in contrary to older children (between 18 months and 4 years old), with an odds ratio of 0.3. [7] Therefore, it is of utmost importance that healthcare professionals are able to differentiate between non-accidental trauma and accidental trauma. Particularly, if the child has an isolated femur fracture, the orthopaedic or paediatric surgeon will probably be the only doctor involved in the treatment.

As previously stated, a femur fracture can in young children be an indicator of non-accidental trauma.

An important concept of forensic medicine is to dissociate extraneous context while investigating and judging information. Experts’ decision should be based on task-relevant information, in order to make an unbiased interpretation of the presented findings. [8] Likewise, dissociation of nonessential context is vital in the diagnostic process of possible non-accidental trauma, to prevent diagnostic errors. Dror et al. highlighted that emotional context and irrelevant context biases experts and non-experts in their judgement on fingerprint identifications. [8] Erroneous identification of medical findings influences the diagnosis and may change the healthcare worker’s decision-making. In order to avoid contamination of the objectivity of findings, we have to identify these cognitive errors. As hypothesized above, erroneous information may influence healthcare professionals in their judgement on medical findings within the diagnostic process of non-accidental trauma of femur fractures in young children. In this study, we asked the participants specifically whether or not the findings on the radiograph had additional evidentiary value in their judgement of the possibility of non-accidental trauma. Hence, we were interested in their judgement on the value of the medical findings itself, not in their judgement on child abuse as diagnosis of the case. The aim of this study was to investigate how, and to which degree, contextual information influences the judgement on the evidential value of medical findings by healthcare professionals.

## Methods

### Study design

Nine clinical vignettes of young children (0–2 years of age) with femur shaft fracture were designed and presented in an online survey with the help of SurveyMonkey (SurveyMonkey Inc., San Mateo, CA, USA). For this anonymized radiographs of children with diaphyseal femur fracture treated in the Paediatric Surgical Centre of Amsterdam UMC, the scope of the study was The Netherlands. We divided the radiographs as follows: 3 transverse, 3 oblique and 3 spiral femur fractures. The participants did not receive any prior information about the type of fracture. For each radiograph, two different possible clinical histories (vignettes) of contextual information were designed: in a first instance, non-accidental trauma was more likely the cause of the fracture (abuse context; A) and in another instance, the accidental trauma was more likely (non-abuse context; B). Risk factors used in the vignettes were weighed and the difference amount of risk factors for non-accidental trauma versus accidental trauma in the two vignettes had to be at least two (available in e-supplement).

An example vignette was shown to the participants to introduce the questionnaire. To avoid influencing the interpretation on femur fractures, a chest radiograph with rib fractures was used in this example. One of the two vignettes with contextual information was randomly assigned to the participant. Each vignette was followed by this question: What value does this radiograph add to your interpretation of the probability of child abuse as a cause of this fracture?

**Fig. 1:**
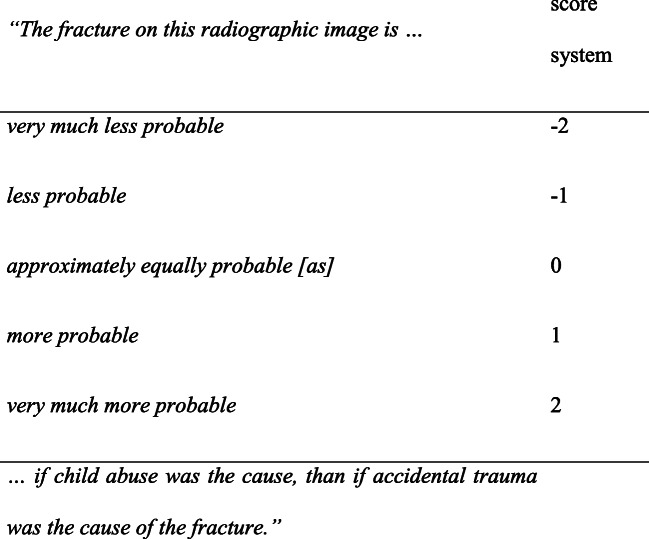
5-point scale with score system, based on the likelihood ratio. A lower score (− 2) points more towards an accident; a higher score (2) points more towards non-accidental trauma

The participants had to answer the question using a 5-point scale, which represents a verbal expression of the diagnostic value or ‘likelihood ratio’ of the evidence (Fig. 1). In other words, participants reported how much more likely the fracture was caused by considering two hypotheses, non-accidental cause versus accident. We asked the participants to value the evidential strength of the fracture itself, rather than focusing on the context given or the diagnosis of the case. In Bayesian terms: We asked for the likelihood ratio of the fracture (given abuse and non-abuse as hypotheses), and not the posterior odds of abuse. This is a typical way of interpreting evidence in forensic science. [9]

### Participants

Eligible participants were medical residents and staff members of the following specialties from various hospitals in The Netherlands: paediatrics, paediatric intensive care unit, paediatric radiologists or radiologists with a special interest in paediatric radiology, (trauma) surgery, paediatric surgery, paediatric orthopaedic surgery and the emergency department. We excluded participants from child protective services (*n* = 3), anaesthesiology (*n* = 11), general practitioner in training (*n* = 1) and if the function was unknown (*n* = 1). These groups were excluded from the analyses due to the fact that the level of response was not sufficient and/or they were not involved in the daily care of children with femur fractures. The responses of fellows were merged with staff responses. Participants were recruited in person, via e-mail and with the help of national professional associations. Participation was on a voluntary and anonymous basis. Participants were neither punished nor rewarded with regard to their answers.

### Secondary outcomes

Secondary outcomes show the difference in level of influence between staff members and residents, different specialties and the relationship between years of experience and level of influence. To determine these outcomes, the participants were asked to answer questions about their current function (staff/resident), years of experience and current specialty.

### Statistical analysis

In order to analyze reported participants’ estimations and interpret the value of contextual information, we used a score system (Fig. 1). The reported values highlighting the strength of evidence of participants are based on the following score system: a positive figure points more towards non-accidental trauma as a cause of the fracture and a negative figure more towards accidental trauma. All the results, tables and figures are based on the abovementioned score system.

The data was analyzed with the generalized linear mixed model procedure of SPSS 24 (IBM Corp., Armonk, NY, USA), taking into account that the reported values were obtained from 172 participants (*N* total response = 1548), as well as the fact that the dependent variable (i.e. the estimate of the evidential strength) is ordinal. The reported evidential strength is expressed as mean with 95% confidence interval. A *p* value < 0.05 was considered statistically significant.

## Results

In total, 172 participants completed the survey. The baseline participant characteristics are depicted in Table 1. Based on a total of 172 participants, 73 (42%) were male. The average years of experience for all staff members were 10.6 years (SD ± 7.7) and 4.1 years (SD ± 2.9) for residents.

**Table 1:** Baseline characteristics of participants

Department	Gender			Experience (years) (mean, SD)
	Male (*n*, %)	Female (*n*, %)	Total (*n*)	
Paediatrics and intensive care unit	16 (26)	45 (74)	61	8.7 (7.4)
Staff	8 (24)	26 (77)	34	12.1 (7.9)
Resident	8 (30)	19 (70)	27	4.3 (3.5)
(Paediatric) Radiology	9 (60)	6 (40)	15	7.1 (5.6)
Staff	6 (55)	5 (46)	11	8.6 (5.8)
Resident	3 (75)	1 (25)	4	2.8 (1.0)
(Trauma) Surgery	26 (70)	11 (30)	37	10.1 (8.1)
Staff	17 (85)	3 (15)	20	14.4 (8.7)
Resident	9 (53)	8 (47)	17	5.18 (2.9)
Emergency department	14 (33)	29 (67)	43	4.5 (3.5)
Staff	7 (29)	17 (71)	24	5.6 (4.1)
Resident	7 (37)	12 (63)	19	3.1 (1.7)
Paediatric/orthopaedic surgery	8 (50)	8 (50)	16	11.7 (7.8)
Staff	8 (50)	8 (50)	16	11.7 (7.8)
Resident	-	-	-	-

**Fig. 2:**
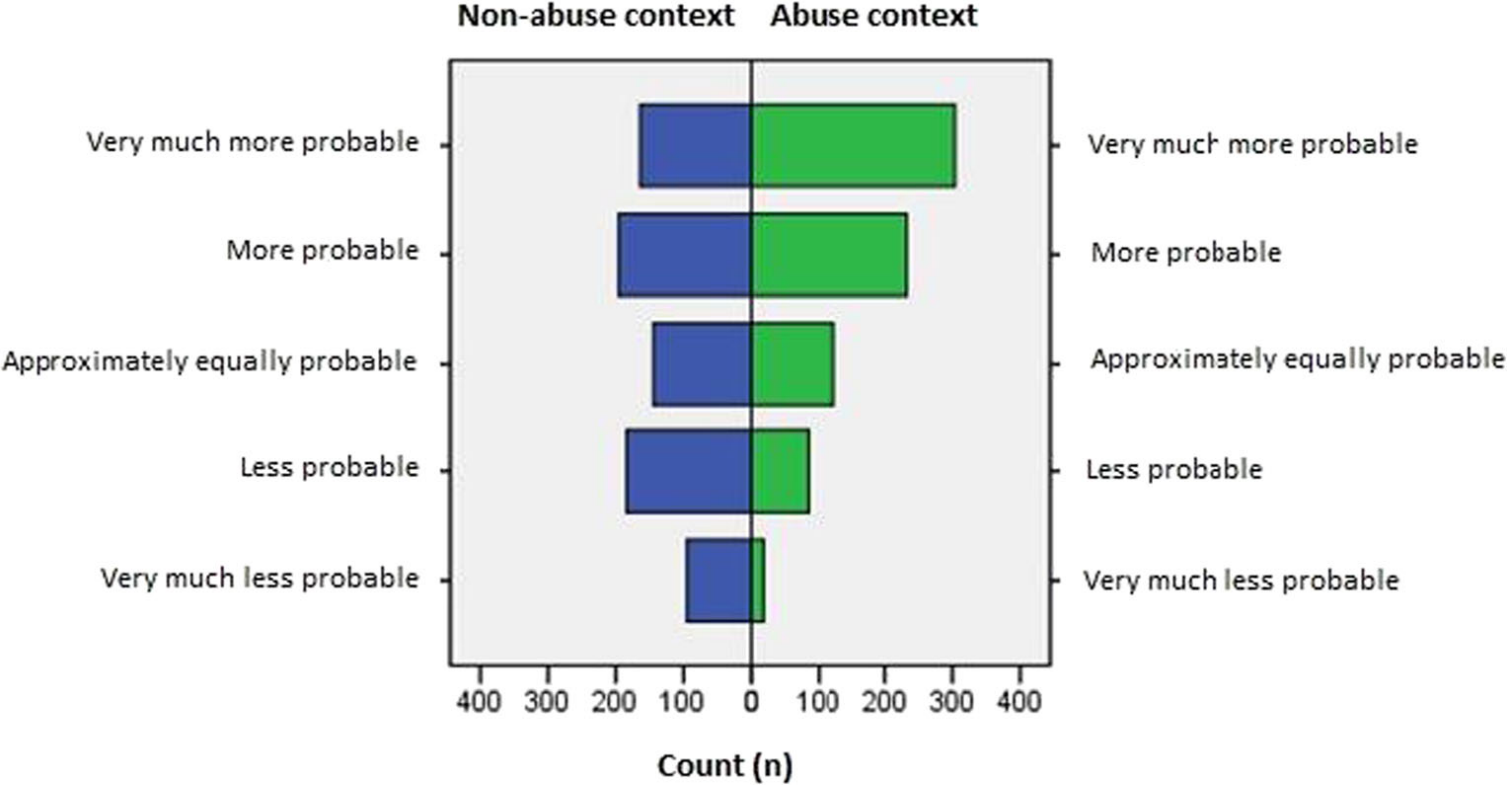
Provided evidential strength (total count) given by participants

**Fig. 3:**
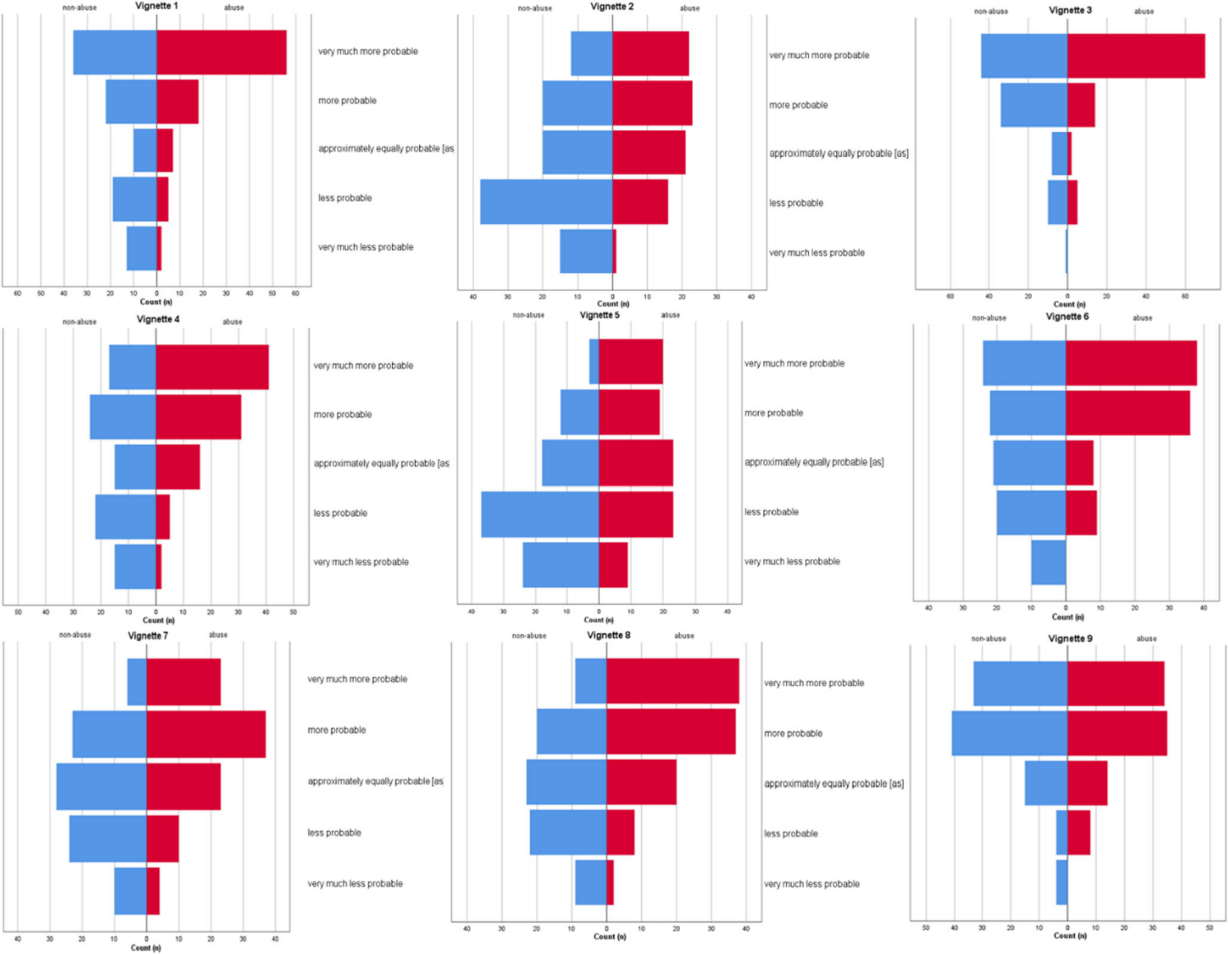
Reported evidential strength of all individual vignettes (total count (*n*))

The vignettes’ overall reported evidential strength with a non-abuse context was 0.19 (*n* = 784; 95%CI 0.10–0.28) and for the abuse context 0.94 (*n* = 764; 95%CI 0.86–1.02). The differences between the reported evidential strength of the non-abuse and abuse context are shown in Fig. 2. There was a significant effect of contextual information on the evidential strength between these two groups (*p* < 0.001). The reported evidential strength of the individual nine vignettes is shown in Table 2 and Fig. 3.

**Table 2:** Reported evidential strength of all vignettes (mean with 95%CI)

Vignette, fracture	Abuse context	Non-abuse context
	Mean (95%CI)	Mean (95%CI)
1. Spiral	1.38 (1.20–1.60)	0.42 (0.11–0.73)
2. Transverse	0.58 (0.33–0.83)	− 0.27 (− 0.52 to − 0.02)
3. Oblique	1.62 (1.45–1.79)	1.17 (0.96–1.38)
4. Spiral	1.02 (0.81–1.23)	0.03 (− 0.26–0.32)
5. Transverse	0.14 (− 0.13–0.41)	− 0.71 (− 0.93 to − 0.49)
6. Oblique	1.11 (0.90–1.32)	0.26 (− 0.02–0.54)
7. Transverse	0.64 (0.41–0.87)	− 0.13 (− 0.37–0.11)
8. Oblique	0.91 (0.70–1.11)	0.01 (− 0.42–0.44)
9. Spiral	1.04 (0.84–1.24)	0.94 (0.72–1.16)

### Secondary outcomes

Participants had a range of 0 to 33 years working experience in their respective specialties. Experience in years of practice did not have a significant effect on the level of influence of contextual information on the evidential strength, as tested by the interaction between the two variables (*p* = 0.49). There was no significant interaction between influence by contextual information and current function (staff versus resident) (*p* = 0.71).

There was a significant difference between the reported evidential strength of female and male participants (*p* = 0.005). Female participants did have a higher base rate towards non-accidental trauma, mean 0.30 (95%CI 0.21–0.39), versus male participants, 0.04 (95%CI − 0.07–0.15); participants reported stronger evidence towards non-accidental trauma in both types of the vignettes compared with male participants. Male and female participants were equally influenced by contextual information, as tested by the interaction between these variables (*p* = 0.81). Mean differences of reported evidential strength of the non-abuse and abuse context were 0.74 and 0.78 for female and male participants, respectively.

**Fig. 4:**
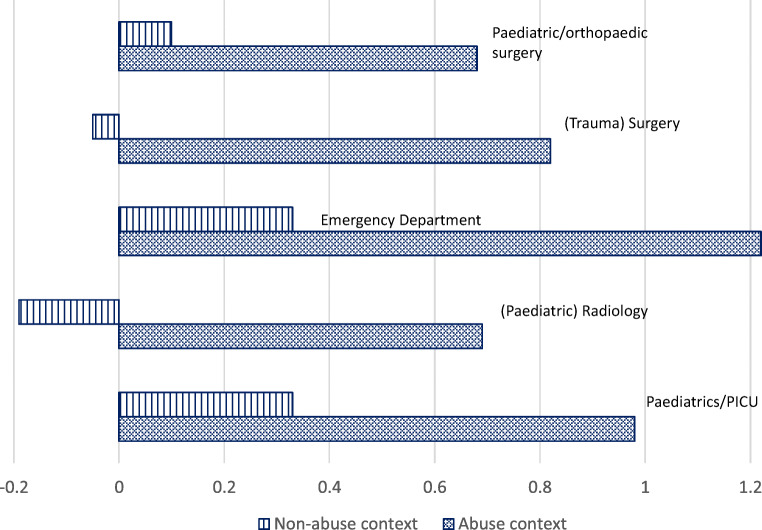
Reported evidential strength per specialty (based on mean scores)

The level of influence by contextual information was not significantly different between specialties, as tested by the interaction between these variables (*p* = 0.40); all specialties were equally influenced by contextual information. Participants from the emergency department (non-abuse 0.33 95%CI 0.17–0.49 versus abuse 1.22 95%CI 1.09–1.35) and paediatric department (non-abuse 0.33 95%CI 0.18–0.48 versus abuse 0.98 95%CI 0.85–1.11) had a higher base rate than the other three specialties. They reported in both types of vignettes stronger evidence towards non-accidental trauma as cause of the fracture (Fig. 4); we can draw the same conclusion even after correcting for gender. The group of (paediatric) radiologists had the lowest base rate (non-abuse − 0.19 95%CI − 0.55–0.17; abuse mean 0.69 95%CI 0.40–0.98). For an overview of the means of all specialties, please refer to Fig. 4.

The nine vignettes were divided by ‘fracture type’, i.e. group based on fracture morphology: spiral (numbers 1, 4, 9), oblique (numbers 3, 6, 8) and transverse (numbers 2, 5, 7). Participants reported a significant difference between the estimates of evidential strength on the three fracture groups (*p* < 0.001). Participants who were assigned to a non-abuse vignette reported stronger evidence towards an accidental trauma as cause of the fracture when it was transverse (Table 2). There was no significant interaction between the level of influence and the fracture group types (*p* = 0.73); in every fracture group, participants were equally influenced by contextual information.

## Discussion

Our study provides evidence that irrelevant context, such as low income, single-parent family households and migrant status, can inappropriately influence healthcare professionals when interpreting the evidentiary value of radiographic findings of femur fractures in young children. In our study, the perception of the evidential strength of the findings changed when a different context was provided to participants. Participants reported stronger evidential strength towards non-accidental trauma as a cause of the fracture when assigned a vignette with an abuse context, rather than when they were provided with a non-abuse context. The core principle of this study is that when a context is given, professionals are influenced (regardless of what the specific context contains) both for making the diagnosis (which is a good one) and the evidential power of the physical findings (which is bias).

In a publication by Croskerry, an overview of the different forms of cognitive errors in healthcare has been presented. [10] In our study, we found that clinicians could either be affected by confirmation bias, i.e. they tended to look for confirming evidence to support a diagnosis and/or by a framing effect, in other words the way they assessed the fracture was influenced by the way in which the case was framed. Our study shows why healthcare professionals should appreciate the impact of diagnostic errors and cognitive errors in medicine and especially refute the inevitability of cognitive diagnostic errors, confirmation bias and the framing effect (how diagnosticians see things may be strongly influenced by the way in which the problem is framed). If context, history and risk factors are not separated from the evidential power of physical findings, then physicians tend to adjust both the prior odds of abuse and the likelihood ratio of the physical findings. This is causing over interpretation of physical findings, leading to contextual bias. A perfect example is the over interpretation of white, two-parent households in missed cases of abusive head trauma, while black, single-parent households were more common in the diagnosed cases [11].

### Secondary outcomes

The impact of contextual information was observed regardless of the level of work experience. There was no interaction between influence by contextual information and an (increased) amount of years of experience. We conclude that increased experience does not prevent influence on conclusions caused by contextual information. Thompson et al. reported similar findings and stated that there was no correlation between increased experience in terms of years of practice and improved intra-observer or inter-observer reliability.[6] In order to prevent the influence as much as possible, it is necessary that clinicians are aware of this vulnerability and not assume that experience prevents from making such assumptions. It is important to implement a procedure that explicitly controls information flow (Contextual Information Management).[12] Furthermore, it is important for healthcare professionals to be receptive to learn from diagnostic and cognitive errors such as the influence of contextual information on medical findings in order to prevent diagnostic errors.

Participants of all specialties were influenced by contextual information. Nevertheless, there were differences between the five specialties intrinsic to this study. Emergency department physicians reported stronger evidential strength towards non-accidental trauma; however, this was the case in both types of vignettes. They had a higher base rate, i.e. emergency department physicians were not more likely influenced by contextual information than participants from other specialties. This may be explained by the ‘nature of their work’ in the emergency department. They have to decide whether to admit, refer or send children home. Hence, they may be more cautious when making decisions in order to prevent sending home potentially abused children. Radiologists had the lowest base rate, and they reported more neutral and less divergent evidential strength than participants from other specialties; however, they were equally influenced by contextual information, possibly because radiologists are more trained to interpret radiographs of femur fractures with a minimum of contextual information. Similar findings were reported by Anderst et al. [13]. In their simulation study, they showed that paediatricians and nurse practitioners often have difficulty differentiating between non-accidental and accidental trauma. In contrast to other studies, they showed an over-diagnosis regarding non-accidental trauma in their study and they identified specific knowledge gaps such as fracture analysis. Only 58% of all participants were able to identify the correct fracture morphology, 73% in low-risk cases and 22% in high-risk cases. [13]

The lowest suspicion for non-accidental trauma was reported in vignette 5. This was a transverse fracture which fitted the trauma mechanism, a fall of great height. The difference between scenarios A and B was the presence of three risk factors in one of the scenarios. These were a single parent, stepsiblings and a low socio-economic status. Scherl et al. reported that the amount of transverse and spiral fractures were equal to 207 young patients diagnosed with non-accidental trauma. [14] However, the perception of most healthcare professionals remains that transverse fractures, in contrast to spiral fractures, are less suggestive for non-accidental trauma. This could explain why participants who were assigned to a non-abuse context in vignette five reported stronger evidential strength towards accidental trauma. In two other vignettes with transverse fractures (vignettes 2 and 7), the participants reported a comparable evidential strength. Of these three vignettes, reported evidential strength of the participants was the lowest among all abuse contexts. This implies that participants had the least suspicion of non-accidental trauma in children with transverse fractures.

The highest suspicion for non-accidental trauma was raised in vignette 3. The difference between A and B for this scenario was the presence of two risk factors in one of the scenarios. These were the fact that the parents were divorced and that one of the parents had a new partner. Children’s age and level of development are main indicators to consider, in order to detect suspicion for non-accidental trauma. [3] An explanation for the high reported evidential strength in vignette 3 can be the pre-ambulatory age—approximately 4 months—of the child in both stories with contextual information. In vignette 9, participants assigned to a story with a non-abuse context reported strong evidential strength towards non-accidental trauma as cause of the fracture. The difference between A and B for this scenario was the presence of two risk factors in one of the scenarios. These were the fact that the parent has a migrant background and that there was no support as regards care for the children. This was contrary to our expectations and to the outcomes of vignettes 1 to 8. In these other vignettes, the participants reported weaker evidential strength towards non-accidental trauma as cause of the fracture, when they were assigned to a non-abuse context. This implies that in vignettes 1 to 8—was expected—participants were more likely to be influenced by the contextual information of the abuse context than of the non-abuse context.

In order to minimize the risk of contextual influence as much as possible, radiologists should be given contextual information with additional care, taking into account whether the information is relevant or not for the specific task. Information regarding the trauma mechanism, age and the child’s development should be given, since these factors are relevant. Radiographs of femur fractures can be used to relate the trauma mechanism and strength to the fracture morphology. Radiologic-forensic correlation of the fracture with the trauma mechanism is a very valuable in addition to all other relevant information. The radiograph is especially relevant if the strength and reported trauma mechanism do not suit the fracture morphology. However, one might question the relevance of the information for a given task. It could therefore be worthwhile to provide this information at a second stage, after a first objective assessment of trauma. Such a procedure fits within a Contextual Information Management procedure and has been applied, for instance, in forensic DNA examination. [15, 16]

### Strengths and limitations

Our study has a number of strengths. First, the participants who responded to our survey were varied; they were staff members and residents from five different specialties from hospitals in The Netherlands. They routinely interpret radiographs of paediatric femur fractures. With a total number of 172 participants, the sample size of this study is relatively large so the statistical power is sufficient to detect small to medium effects. Second, this study used existing histories in the vignettes, i.e. the vignettes show a significant reflection of cases that are treated on a daily basis. Usually, studies on traumatic injuries relating to non-accidental trauma neglect the influence of contextual information. Third, to prevent bias of fracture morphology in the survey, participants did not receive any prior information about the fracture types of the radiographs. Fourth, this study shows the influence of context, of which occurrence is not inevitable, an important cognitive error that needs more attention in the decision-making process regarding non-accidental trauma and should be reduced using appropriate measures during this process.

A limitation of our study is that we did not design a radiograph without a context as a ‘baseline’ measurement. Therefore, we cannot know whether the provided evidential strength differs from a baseline measurement without any context. Participants who were assigned to a non-abuse context reported an evidential strength close to zero, which indicates that they thought that the cause of the fracture was equally probable for non-accidental versus as for accidental. Therefore, we assume these answers are close to a baseline without any contextual information.

Unfortunately, we did not ask the participants whether they would initiate a work-up for suspected non-accidental trauma. This should be addressed in future research, because it would be interesting to know whether the difference in perceived evidential strength results in a difference in clinical behavior, i.e. whether healthcare professionals would initiate a work-up. Due to the lack of ‘sentinel’ characteristics of femur fractures, the children’s youth is the most important indicator of non-accidental trauma. [1, 3–5] Therefore, every young child should get a work-up conform to the AAOS Clinical Practice Guidelines for Pediatric Femur Fractures, endorsed by the EPOS [17], regardless of fracture morphology or reported trauma mechanism. At last, we requested the participants to indicate the evidential strength of the specific fracture. The evidential strength of the fracture is, and should be, independent of task-irrelevant contextual information. However, most healthcare professionals are not familiar with indicating the evidential strength of medical results. We cannot exclude that some healthcare professionals may have misinterpreted the question. They may have reported the probability of non-accidental trauma for each vignette instead of the evidential strength of the fracture itself (which should be ‘neutral’, because the fracture morphology does not differ between accidental or non-accidental causes). Context can be a considerable part of the ‘diagnosis’ of non-accidental trauma. Although we cannot determine to what extent participants misinterpreted the question, this does not negate the adverse effect of contextual information on the interpretation of medical results. The determination of the evidential strength on medical results appears to be influenced by context regardless.

## Conclusion

When assessing radiographs of fractures in young children, healthcare professionals are influenced by irrelevant contextual information and it leads them to a discriminatory bias against certain groups based upon the families’ level of income, marital status or migrant status. Although the various specialists reported a different degree of evidential strength, all specialties were significantly influenced by contextual information; however, emergency department and paediatric doctors were most likely to conclude that non-accidental trauma was the cause, versus paediatric radiologists who reported least likely non-accidental trauma. Same results were found when focusing on the influence of gender; women reported a stronger evidential strength towards non-accidental trauma in comparison with men. It is doubtful whether influence of contextual information can be prevented completely; however, healthcare professionals should appreciate the impact of cognitive errors can have an (potential) influence on their decision-making, ergo causing diagnostic errors. Although it is important to prevent this as much as possible, recognition of its existence is a first step in this process, taking into account that more experience does not protect against this influence. We therefore recommend that in future research and clinic, the awareness of the influence of contextual information will be addressed and whether this leads to differences in behavior of healthcare professionals regarding a work-up. Furthermore, a Contextual Information Management procedure should be implemented in the diagnosing process of non-accidental trauma.

## Electronic supplementary material

ESM 1 (DOCX 241 kb)

## Abbreviations

CAN: Child Abuse and Neglect team
CI: Confidence interval
ED: Emergency department
NAT: Non-accidental trauma
SD: Standard deviation
