# Supplementary material for: Paediatric femur fractures—the value of contextual information on judgement in possible child abuse cases: are we bias?
Source: Eur J Pediatr. 2020 Jun 17;180(1):81–90. doi: 10.1007/s00431-020-03704-6 (PMC7782380; doi:10.1007/s00431-020-03704-6)
Supplement: Supplementary file 1 — (DOCX 241 kb) [file 431_2020_3704_MOESM1_ESM.docx]

**E-Supplement**

**Clinical Vignettes:** 9 radiographs (3 transverse, 3 oblique and 3 spiral fractures) with a plausible story of contextual information. For each radiograph, 2 different stories of contextual information are designed: in one child abuse will be more likely as a cause of the fracture (group A) and in the other case an accidental trauma (group B).

| **#** | **Type of fracture** | **Group** | **Story of contextual information** |
| --- | --- | --- | --- |
| 1. | Spiral | A | A boy of 20 months old visits the emergency department together with his parents. They have been living in the Netherlands for two years. The mother explains that he had stumbled over his own feet while he was running. It happened at day care. The boy is crying, seems to be in a lot of pain and makes a frightened impression. His thigh is swollen and painful. His father is very upset because of the long wait ‘just like last year’. They visited the ED because of a humeral fracture of this boy. The father smells like alcohol. |
|  |  | B | A boy of 20 months old visits the emergency department together with his mother. She explains that he stumbled over his own feet while running. It happened at day care. The day care teacher did not witness the fall, but was in the same room while it happened and heard him crying. The patient seems to have a lot of pain and makes a frightened impression. His thigh is swollen and painful. The mother is very concerned. The father stayed at home with the older sister. |

Abuse: N=84, median 2; mode 2; mean 1,38 (95%CI 1.20 – 1.60)


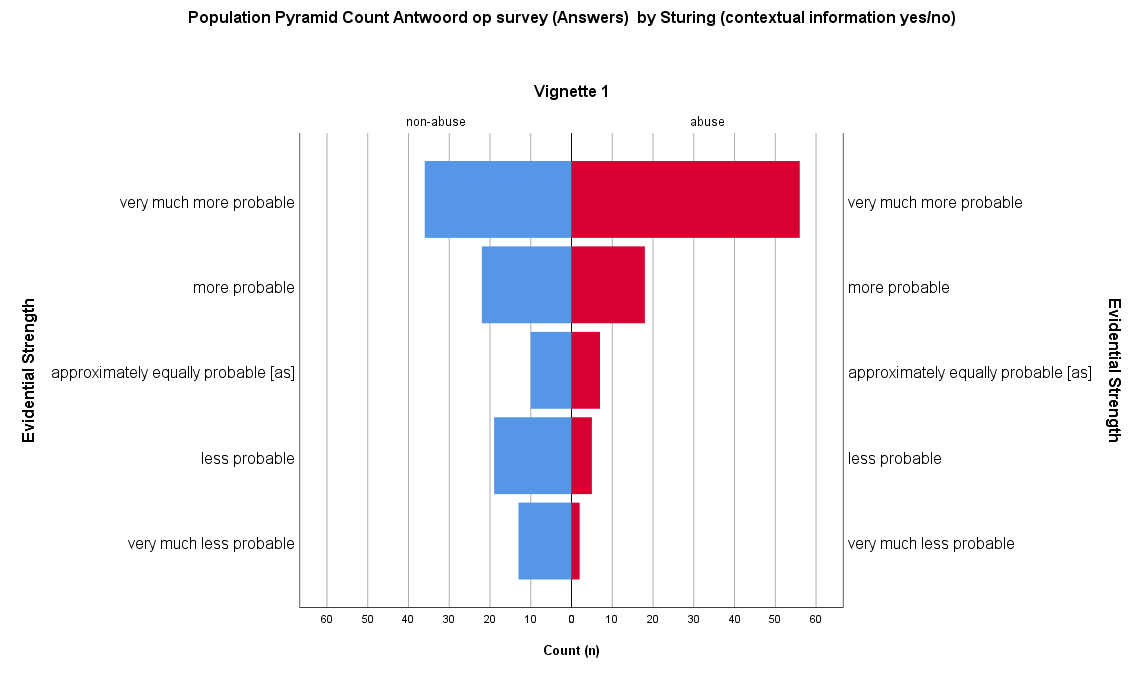
Non-abuse: N=88, median 1,00; mode 2; mean 0,42 (95%CI 0.11 – 0.73)

| **#** | **Type of fracture** | **Group** | **Story of contextual information** |
| --- | --- | --- | --- |
| 2. | Transverse | A | A boy of 6 months old visit the emergency department together with his parents. There is a language barrier, but father tries to explain that he held his son above his head (about 2m high) and accidentally dropped him on the ground. The mother was at that moment shopping with the other three children. The boy is crying a lot and clearly has pain in his thigh. He was premature born, however he develops well. The family is of immigrant origin. |
|  |  | B | A boy of 6 months old visit the emergency department together with his parents. His father explains that he held his son above his head (about 2m high) and accidentally dropped him on the ground. The mother and his sister of 3 years old witnessed the fall. The boy is crying and clearly has pain in his thigh. The father seems to feel very guilty about the accident. |

Abuse: N= 76; median 1,00; mode 1; mean 0,58 (95%CI 0.33 – 0.83)

Non-abuse: N=96; median -1,00, mode -1, mean -0,27 (-0.52 – -0.02)


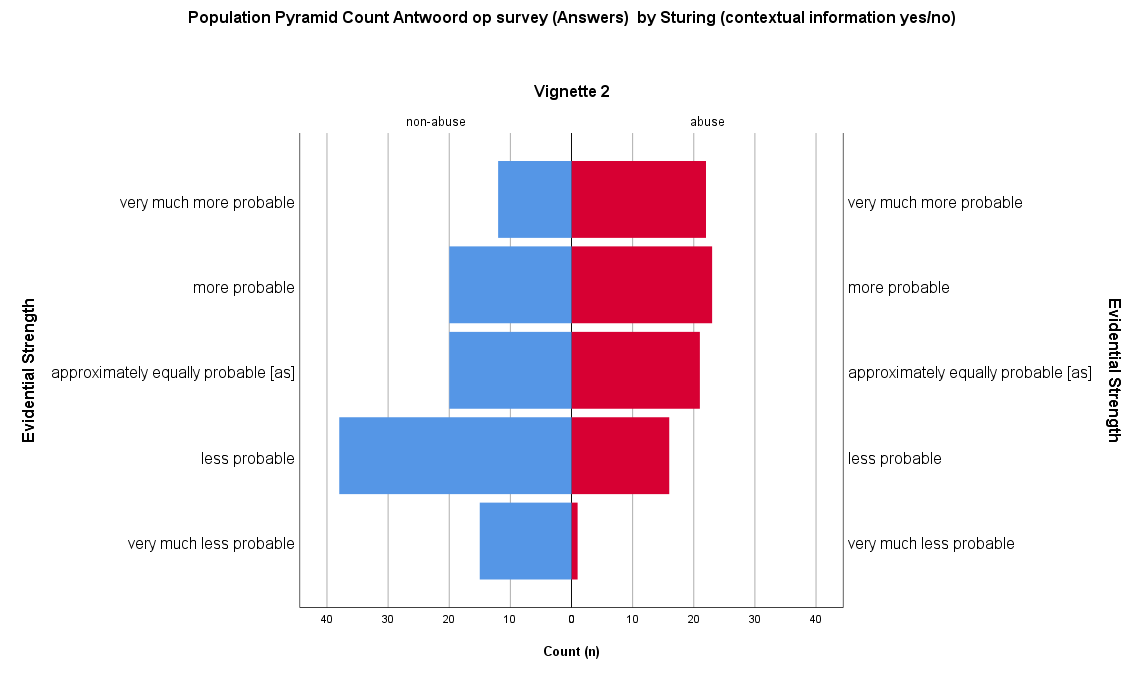


| **#** | **Type of fracture** | **Group** | **Story of contextual information** |
| --- | --- | --- | --- |
| 3. | Oblique | A | A 3 months old girl attends the emergency department with her mother and stepfather. According to the mother the girl started to cry a lot after she woke up from her afternoon nap. Nobody witnessed a trauma, but the mother thinks that her leg may have been trapped in the side bars of the crib. They came to seek medical attention because the girl did not stop crying. Both parents don’t know what have happened. The thigh is swollen and painful. The mother and stepfather live together since 6 months. |
|  |  | B | A 3 months old girl attends the emergency department with her parents. According to the mother the girl started to cry a lot after she woke up from her afternoon nap. Nobody witnessed a trauma, but when the mother took her from the crib, her leg seemed to be trapped in the side bars of the crib and her thigh seemed to be swollen. They came to seek medical attention because the girl did not stop crying, she usually barely cries. The thigh is swollen and painful. Parents are married, this is their first born. |

Abuse: N=85, median 2; mode 2; mean 1,62 (95%CI 1.45 – 1.79)


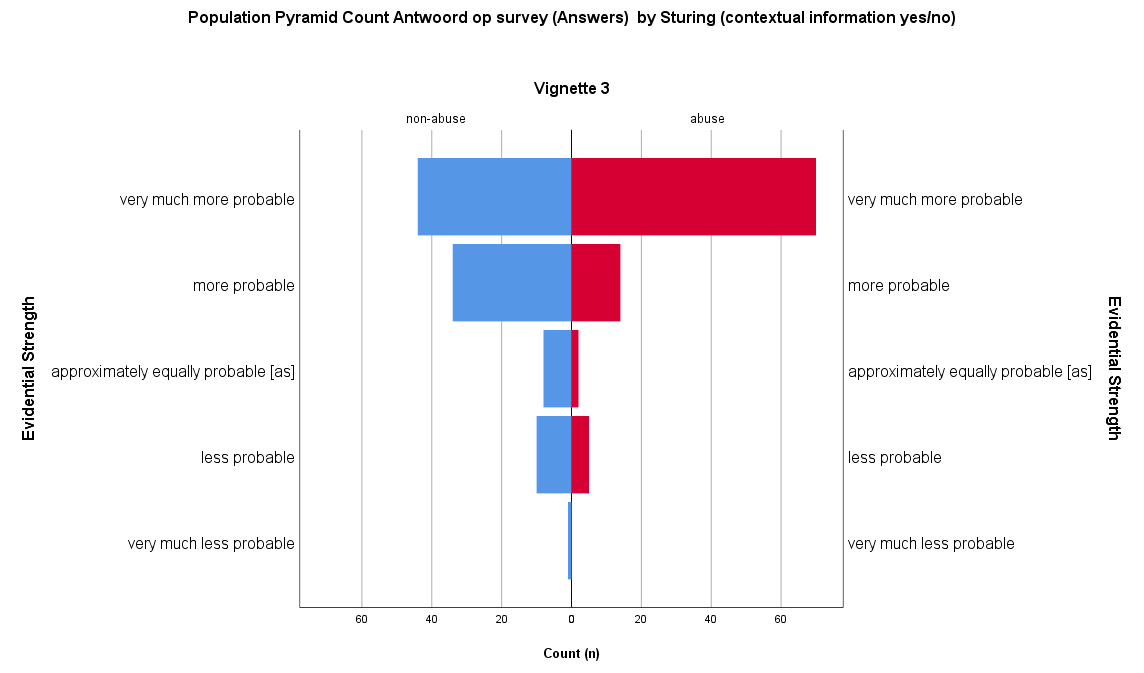
Non-abuse: N=87, median 1,00; mode 2; mean 1,17(95%CI 0.96 – 1.38)

| **#** | **Type of fracture** | **Group** | **Story of contextual information** |
| --- | --- | --- | --- |
| 4. | Spiral | A | A boy of approximately 2 years old is referred to the emergency department. According to the new boyfriend of the mother the boy fell while he was walking backwards at the playground. He fell on his right thigh. There are no other witnesses of the fall, his twin sister was staying at their biological fathers’. The boy suffers from severe asthma, seemed to be anxious during the hospital visit and cries. His right thigh is painful and swollen. |
|  |  | B | A boy of approximately 2 years old is referred to the emergency department. According to the mother he fell while he was walking backwards at the playground. He fell on his right thigh. His little sister was playing in the sandpit. He started to cry immediately, so they went to seek medical attention right away. The boys seemed to be anxious during the hospital visit and cries. His right thigh is painful and swollen. His father is on his way to the emergency department. |

Abuse: N=86; median 1,00; mode 2; mean 1,02 (95%CI 0.81 – 1.23)

Non-abuse: N=86; median 0,00; mode 1; mean 0,03 (95%CI -0.26 – 0.32)


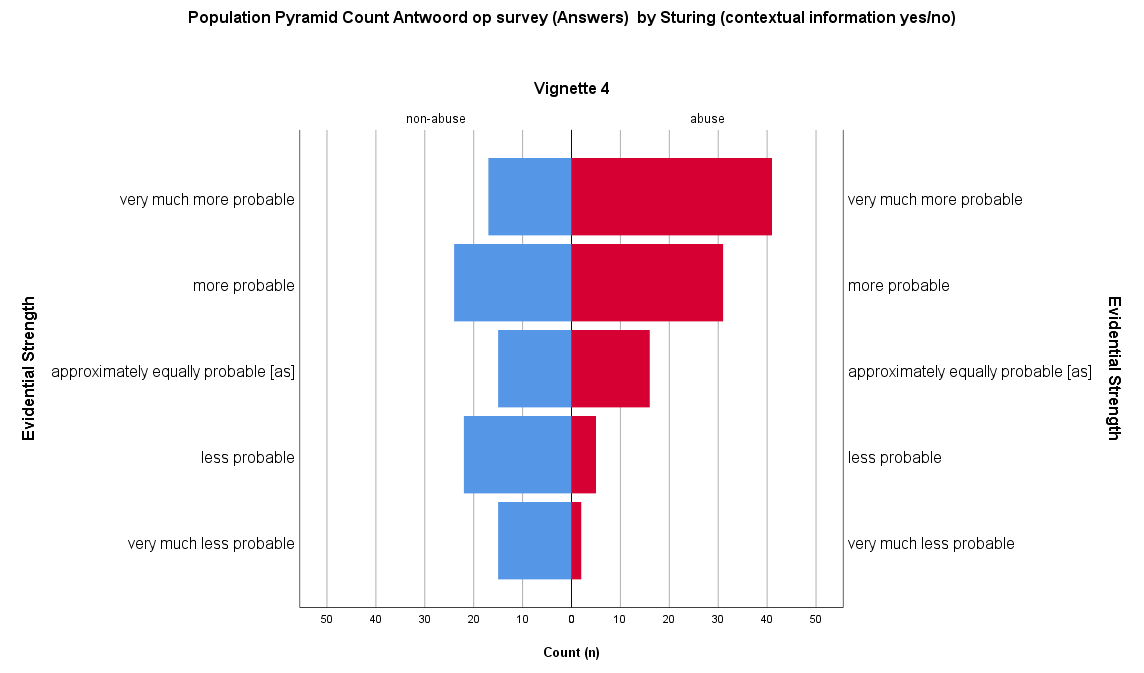


| **#** | **Type of fracture** | **Group** | **Story of contextual information** |
| --- | --- | --- | --- |
| 5. | Transverse | A | A girl of 15 months old attends the emergency department with her neighbor (a friend of her mother). She explains that she fell into the stairwell together with her mother. Because the girl did not stop crying together with her swollen thigh, she came to the emergency department. The mother did not have transport to travel to the emergency department and besides that, she had to stay home with the newborn twins. The neighbor did not witness the fall, but walked outside to check on them when she heard crying. The mother lives with this girl, her two half-brothers and financially they live from a national benefit. |
|  |  | B | A girl of 15 months old attends the emergency department with her parents. The mother explains that they fell together into the stairwell because the mother slipped while she was carrying the girl. The girl did not stop crying and had a swollen thigh. The father did not witness the fall (he was in the kitchen). Both parents are upset and concerned about their daughter. Her sister stays with the neighbor at this moment, she usually babysits the girls when the parents have to work. |

abuse: N=85; median 0,00; mode -1; mean 0,14 (95%CI -0.13 – 0.41)


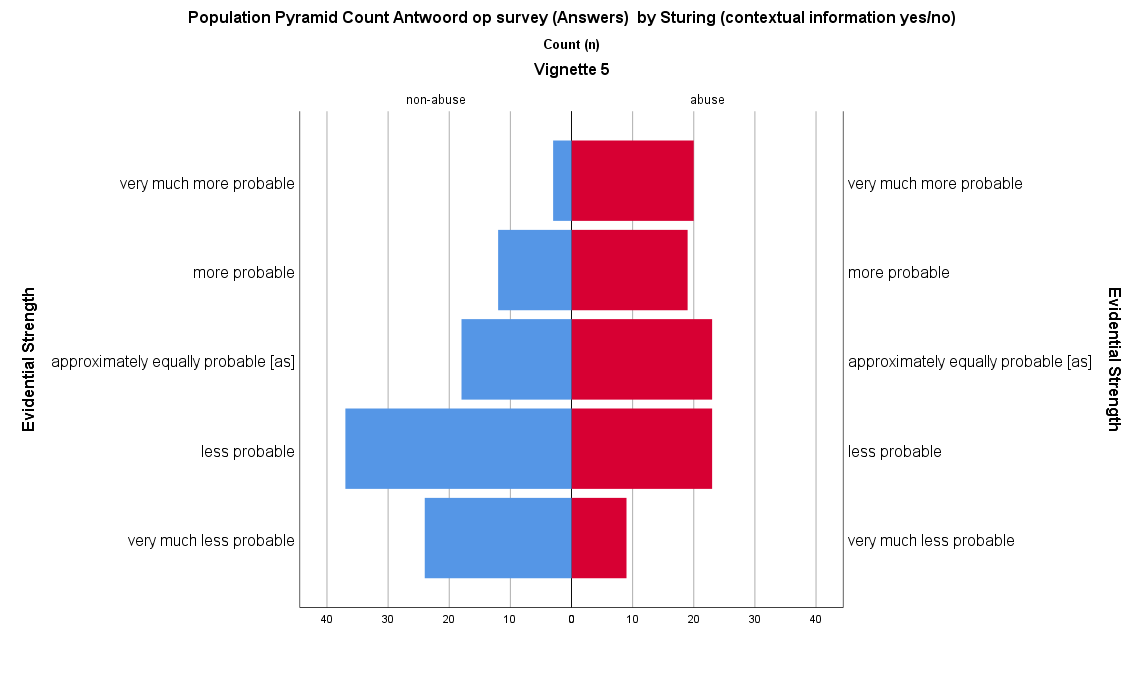
non-abuse: N=87; median -1.00; mode -1; mean -0,71 (95%CI -0.93 – -0.49)

| **#** | **Type of fracture** | **Group** | **Story of contextual information** |
| --- | --- | --- | --- |
| 6. | Oblique | A | A mother visits the emergency department in the evening (7 pm) with her 4 month old son. The single mother explains that they fell together from the staircase. It happened around noon. The mother is not sure how he fell. The boy is crying out loud and his thigh is swollen and has an abnormal position. He was premature born and suffers from upper airway infections. However he develops well. |
|  |  | B | Parents visit the emergency department with their 4 month old son. The mother explains that they fell together from the staircase. It happened in the afternoon. The mother is not sure how he fell. The boy started to cry immediately. She waited for the father to come home (around 30 minutes) and they went to the emergency department. His thigh is painful and in an abnormal position. He had an uncomplicated birth and develops well. |

abuse: N=81; median 1,00; mode 2; mean 1,11 (95%CI 0.90 – 1.32)

non-abuse: N= 91; median 0,00; mode 2; mean 0,26 (95%CI -0.02 – 0.54)


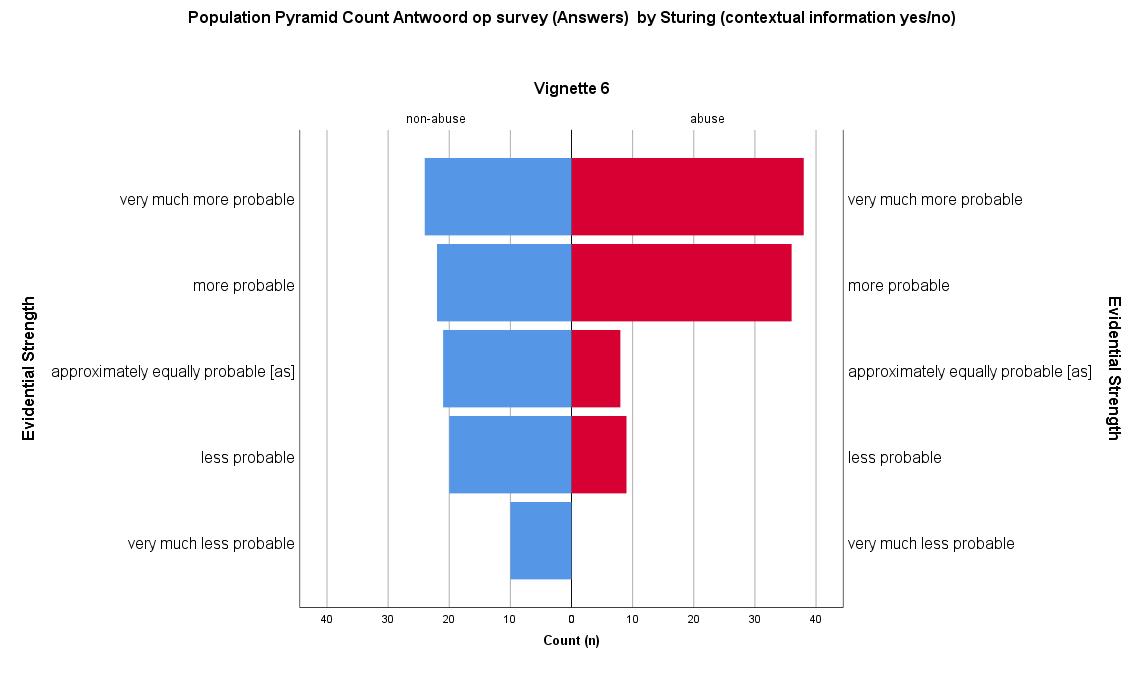


| **#** | **Type of fracture** | **Group** | **Story of contextual information** |
| --- | --- | --- | --- |
| 7. | Transverse | A | A 12 month old boy visits the emergency department with his mother. She tells that her son probably climbed on a chair in the living room (while he was playing) and fell from it on the ground. There are no witnesses, the mother was in the kitchen preparing dinner. His thigh is swollen, painful and in abnormal position. The mother is fulltime at home to raise him, he is her only child. She does not work because of a recent burnout. His father will be admitted to an rehab clinic for his addiction next week. |
|  |  | B | A 12 month old boy visits the emergency department with his mother. She tells that her son probably climbed on a chair in the living room (while he was playing with his sister) and fell from it on the ground. Only his little sister witnessed the fall. The father was at work and the mother preparing dinner in the kitchen. She heard him crying and found him next to the chair. His thigh is swollen, painful and in abnormal position. The father is at home with the little sister. |

Abuse: N=85; median 1,00; mode 1; mean 0,64 (95%CI 0.41 – 0.87)

Non-abuse: N=87; median 0,00; mode 0; mean -0,13 (95%CI -0.37 – 0.11)


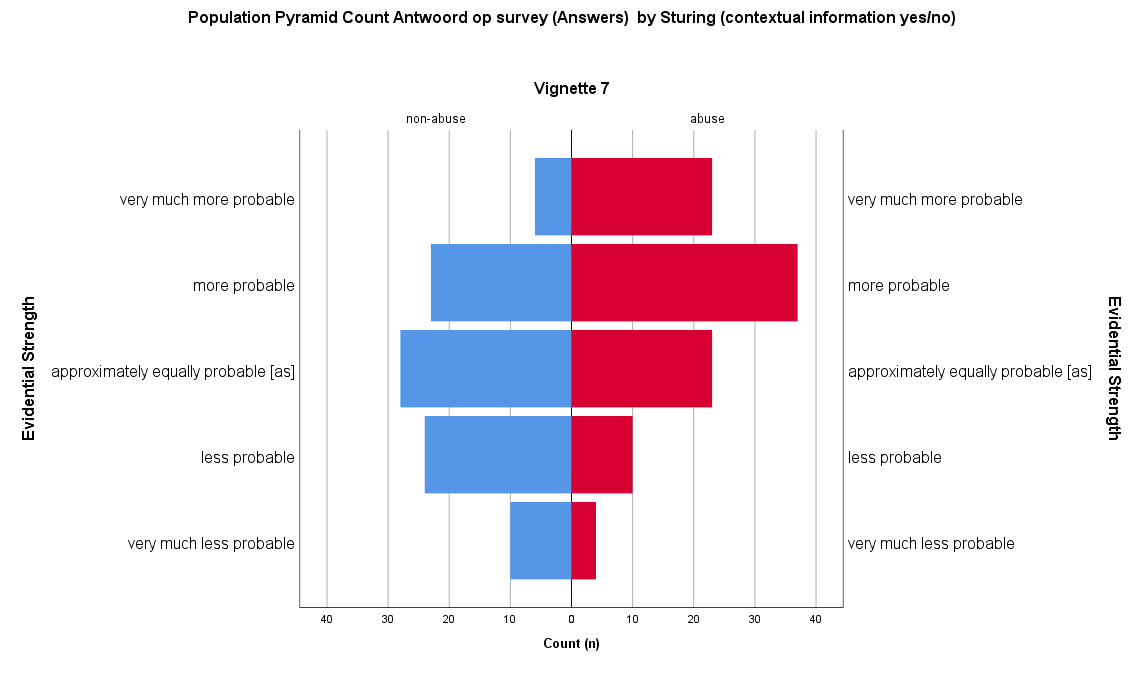


| **#** | **Type of fracture** | **Group** | **Story of contextual information** |
| --- | --- | --- | --- |
| 8. | Oblique | A | A 23 month old girl with Down Syndrome visits the emergency department with her father. He explains that his daughter was playing in the garden and went outside to check on her when he heard her crying. She was lying on the ground. The father thinks that her brother may have pushed her, he can be a little rough. The other brother and mother were not at home. Parents divorced last year and the father is looking for a job. The children live at their mothers’ place. |
|  |  | B | A 23 month old girl with Down Syndrome visits the emergency department with her father. He explains that his daughter was playing in the garden together with her brother. He went outside to check on her when he heard her crying. She was lying on the ground. Her brother told that she fell of the slide (about 1m high). He was the only one who witnessed the fall. The father was cleaning up the kitchen and the mother was at her work. |

Abuse: N=97; median 1,00; mode 2; mean 0,91 (95%CI 0.70 – 1.11)

non-abuse: N=75; median 0,00; mode 0; mean 0,01 (95%CI -0.42 – 0.44)


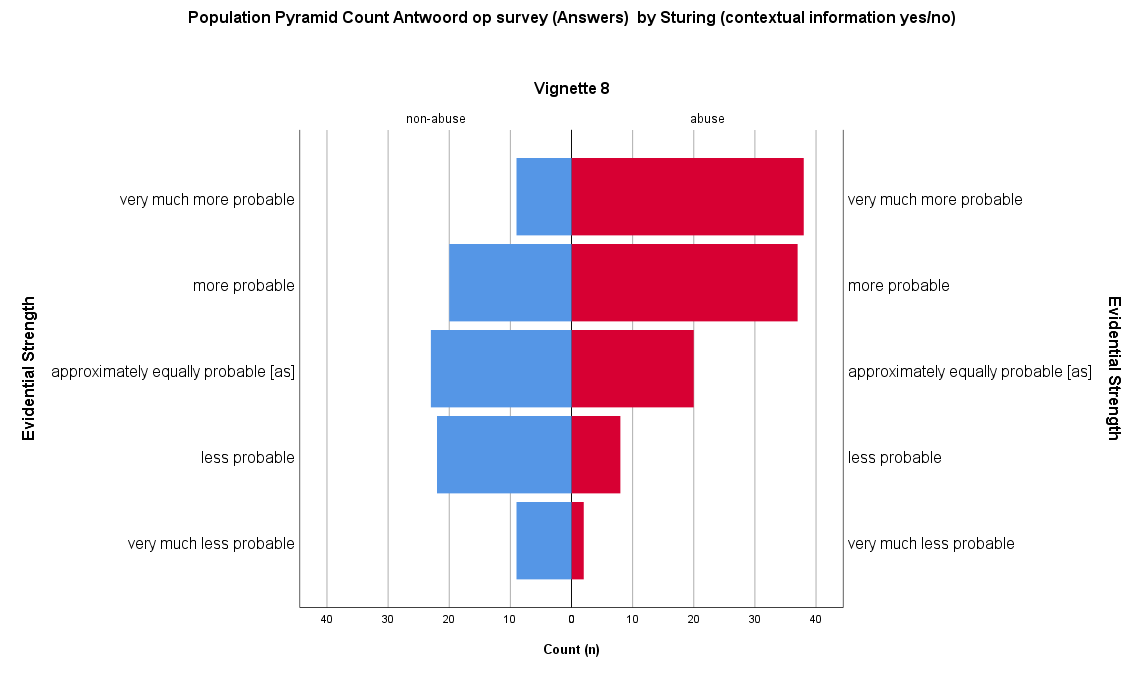


| **#** | **Type of fracture** | **Group** | **Story of contextual information** |
| --- | --- | --- | --- |
| 9. | Spiral | A | A 22 month old boy visits the emergency department with his mother late in the evening. The mother tells that he stumbled over his trousers while walking, approximately around 5.30 pm. He started to cry immediately, she tried to comfort him. His right thigh is swollen and painful with a hematoma. The father passed away three months ago, the family of the mother lives in their country of origin. She has 4 children in total and seems to be tired. |
|  |  | B | A 22 month old boy visits the emergency department with his mother. The mother tells that he stumbled over his trousers while walking, approximately around 5.30 pm. He started to cry immediately, she tried to comfort him and called their family doctor. He referred her to the emergency department. His right thigh is swollen and painful with a hematoma. The mother is very concerned and seems to be tired. The father passed away three months ago, fortunately her family is supporting her. Her parents help to take care of the children. |

abuse: N=85; median 1,00; mode 1; mean 1,04 (95%CI 0.84 – 1.24)


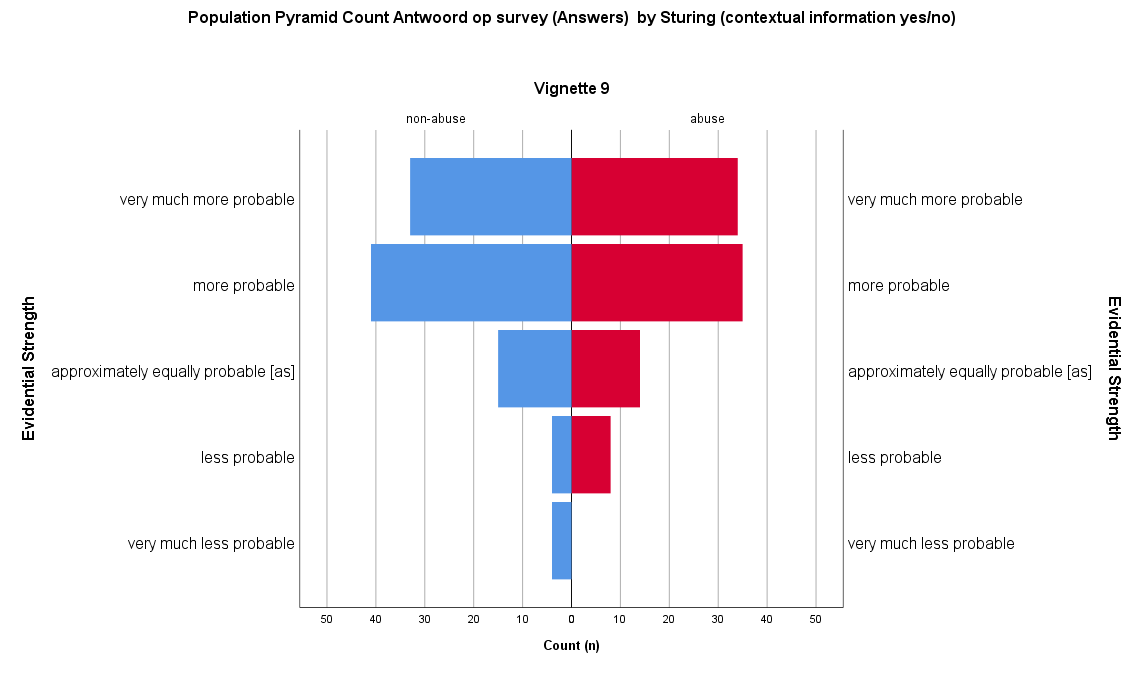
non-abuse: N=87; median 1,00; mode 1 mean 0,94 (95%CI 0.72 – 1.16)
